# Exercise-Based Pulmonary Rehabilitation for Interstitial Lung Diseases: A Review of Components, Prescription, Efficacy, and Safety

**DOI:** 10.3389/fresc.2021.744102

**Published:** 2021-11-16

**Authors:** Renata G. Mendes, Viviane Castello-Simões, Renata Trimer, Adriana S. Garcia-Araújo, Andrea Lucia Gonçalves Da Silva, Snehil Dixit, Valéria Amorim Pires Di Lorenzo, Bruno Archiza, Audrey Borghi-Silva

**Affiliations:** ^1^Cardiopulmonary Physiotherapy Laboratory, Department of Physiotherapy, Federal University of Sao Carlos (UFSCar), Sao Carlos, Brazil; ^2^Cardiorespiratory Rehabilitation Laboratory, Health Sciences Department, University of Santa Cruz do Sul, Santa Cruz do Sul, Brazil; ^3^Department of Medical Rehabilitation Sciences, College of Applied Medical Sciences, King Khalid University, Abha, Saudi Arabia; ^4^Laboratory of Spirometry and Respiratory Physiotherapy, Department of Physiotherapy, Federal University of Sao Carlos (UFSCar), Sao Carlos, Brazil

**Keywords:** physical exercise, rehabilitation, respiratory disease, exercise intolerance dyspnea, interstitial lung disease

## Abstract

Interstitial lung diseases (ILDs) comprise a heterogeneous group of disorders (such as idiopathic pulmonary fibrosis, sarcoidosis, asbestosis, and pneumonitis) characterized by lung parenchymal impairment, inflammation, and fibrosis. The shortness of breath (i.e., dyspnea) is a hallmark and disabling symptom of ILDs. Patients with ILDs may also exhibit skeletal muscle dysfunction, oxygen desaturation, abnormal respiratory patterns, pulmonary hypertension, and decreased cardiac function, contributing to exercise intolerance and limitation of day-to-day activities. Pulmonary rehabilitation (PR) including physical exercise is an evidence-based approach to benefit functional capacity, dyspnea, and quality of life in ILD patients. However, despite recent advances and similarities with other lung diseases, the field of PR for patients with ILD requires further evidence. This mini-review aims to explore the exercise-based PR delivered around the world and evidence supporting prescription modes, considering type, intensity, and frequency components, as well as efficacy and safety of exercise training in ILDs. This review will be able to strengthen the rationale for exercise training recommendations as a core component of the PR for ILD patients.

## Definition, Types, Classification, and Epidemiology of Interstitial Lung Diseases (ILDs)

ILDs comprise a heterogeneous group of disorders that lead to diffusing inflammation and/or fibrosis of the lung parenchyma and vasculature. Classifications of ILDs are based on known (inhaled agents, drugs, infections, etc.) or unknown (idiopathic pulmonary fibrosis—IPF, sarcoidosis, asbestosis and pneumonitis) etiology, specific disease entities, or clinical, histological, or radiological patterns (1). Roughly, one-third of all diagnosed ILDs are a consequence from known exogenous and endogenous causes, whereas two-thirds are idiopathic. Nonetheless, all ILD types share a common pattern of physiological abnormalities, which is inflammation and/or fibrosis of the lung parenchyma, resulting in the presence of dyspnea, decreased pulmonary function, impaired gas exchange, reduced cardiovascular function, and exercise intolerance (2, 3).

According to the Global Burden of Disease Study (4), between the years of 1990 and 2013, there was an 86% increase in ILDs-related years of life lost, and, for the first time, among the top 50 causes of global years of life lost. The estimate for overall ILD prevalence in the United States is ~81 per 100,000 for males and ~67 per 100,000 for females per year (5). Registries conducted in Europe in 2004 involved more than 1,000 cases indicating a prevalence rate of ~17 per 100,000 inhabitants and an incidence rate of 3.62 per 100,000 per year (6, 7). However, more recent registries indicate an even higher incidence of ILDs. For instance, the estimated numbers for a registry conducted in Denmark reaches ~34 per 100,000 person-years (8).

## Pathophysiology, Exercise Limitation, and Functional Impairment

The genesis of ILDs is complex and involves a series of diffuse remodeling of the lung parenchyma due to direct or systemic injury (9). In brief, the persistent inflammatory process caused by injury of the lung tissue creates a cascade of events, causing an increase in reactive oxygen species, growth factors, cytokines, and chemokine release, hence more damage is caused along with tissue fibrosis (10). Based on this, multiple consequences are observed (i.e., alveolar-capillary membrane thickening, decrease of pulmonary gas exchange, dyspnea), ultimately leading to functional impairment and exercise intolerance.

**Figure 1:**
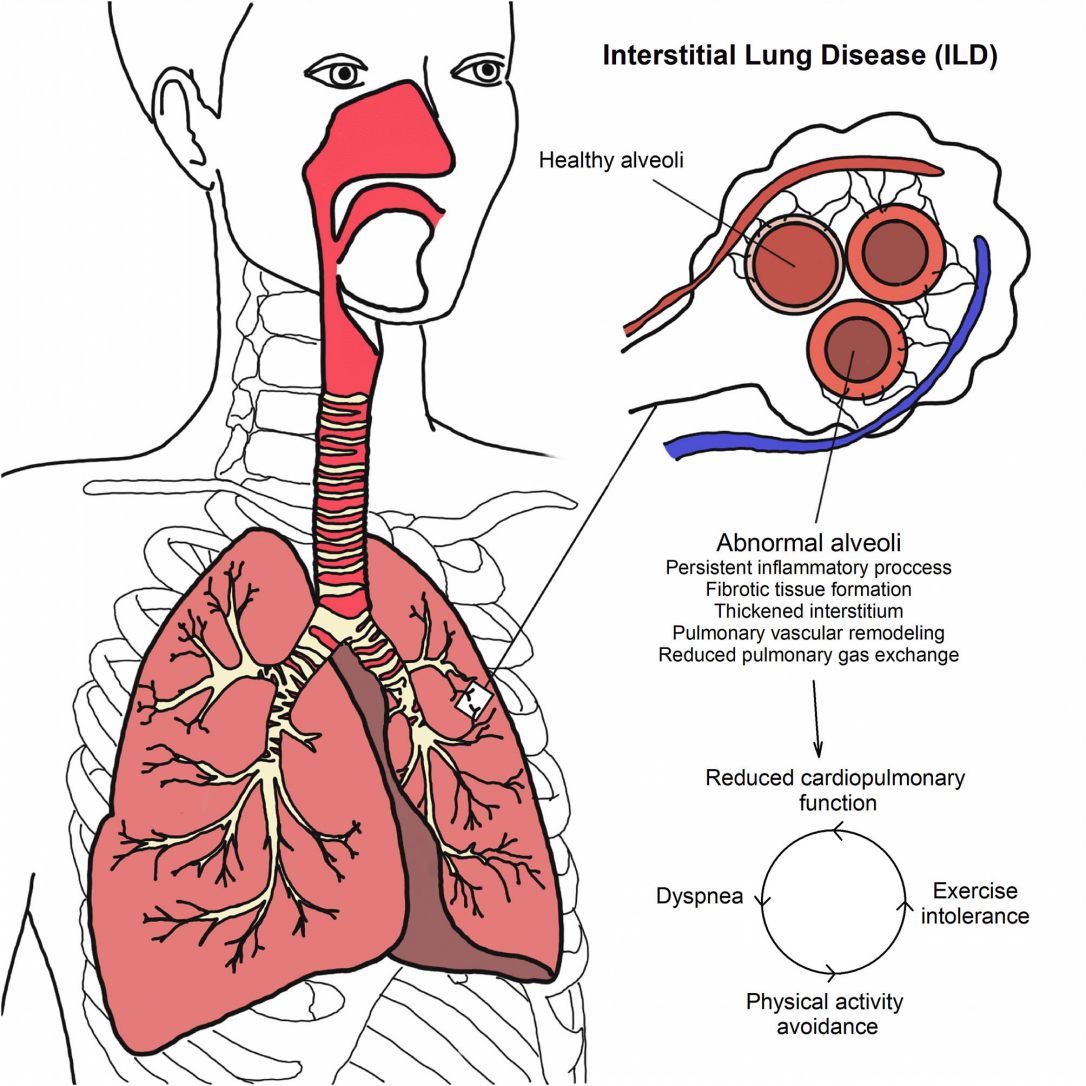
Pathophysiology and cycle of reduced physical activity levels in ILDs.

Due to the persistent inflammatory process and formation of fibrotic tissue, structural and mechanical pulmonary system alterations are observed and considered main causes of a pathological reduction of pulmonary and cardiovascular functions (11, 12). For instance, ILD patients are reported to exhibit reduced static and dynamic lung volumes (2). Moreover, the diffusing capacity is also impaired (2). Collectively, these pathological reductions in cardiopulmonary function contribute to an increase in exertional dyspnea and exercise intolerance; thus, ILD patients tend to avoid situations where they might experience breathlessness, fomenting a cycle of reduced physical activity levels and increased sedentary lifestyle (13) (see Figure 1).

Numerous pathophysiological responses to exercise are observed in ILD patients, such as ventilatory (i.e., abnormal breathing patterns, ventilatory inefficiency, higher oxygen cost of breathing), diffusional (i.e., impaired pulmonary gas exchange, arterial hypoxemia), cardiovascular (i.e., increased pulmonary artery pressures), and skeletal (i.e., reduced muscle strength and resistance to fatigue) dysfunctions, among others. Therefore, each patient with a diagnosis of ILD must be considered as a different and individualized organism as the contribution of each system to exercise intolerance may vary. For a comprehensive review on the topic, we direct the interested reader elsewhere (2).

## Exercise Limitation In ILDs

The importance of evaluating exercise capacity in ILD patients has been investigated more recently, due to a relationship with increased survival (14). The current recommendation indicates that ILD patients have experienced exercise limitation, providing an integration of contributors to functional limitation, disease progression and enrollment in a pulmonary rehabilitation (PR) program (15, 16).

Cardiopulmonary exercise testing (CPET) provides important information concerning exertional dyspnea and mechanisms of exercise limitation as a comprehensive assessment of the physiological changes in the respiratory, cardiovascular and musculoskeletal systems during exercise, which may be also useful for exercise prescription (17–19). Maximum and peak oxygen uptake corresponding to a measurement of the capacity for aerobic activity has been linked to survival in ILD patients (18). As above mentioned, among other factors, the limitation on exercise in these patients may be the consequence of abnormal responses of gas exchange, ventilatory efficiency or ventilatory mechanical limitation (20). Older age and higher systolic pulmonary artery pressure were also independent predictors of poor submaximal exercise capacity in patients with sarcoidosis (21). However, the prognostic value of CPET parameters and responses of ILD patients remains controversial and requires additional research. Exercise tests provide a global assessment of functional capacity and this may be particularly relevant for ILDs with peripheral or respiratory muscle involvement, or where increased symptoms are not explained by changes in respiratory function variables (14).

The 6-min walk test (6MWT) is more commonly used to monitor progress of ILDs over time and during rehabilitation programs (22). Walking covered is an independent and discriminating predictor of mortality among patients with IPF (23). Oxygen desaturation impairment was associated with a 2 fold greater hazard of dying during 6 months of follow-up in IPF. This suggests that changes in the 6MWT may help to assess the costs and benefits of new therapies to individual patients of the test and individual patient characteristics (24, 25).

Peripheral muscle dysfunction is also a critical factor in determining exercise intolerance in patients with chronic lung diseases, including ILD. There are several well-established muscle dysfunction-promoting mechanisms in ILDs (26, 27). Hypoxia is one of the factors that can result in increased oxidative stress and adversely affect muscle performance (26, 27). Guler et al. (28) used a skeletal muscle index (SMI), as the sum of the muscle mass of the upper and lower limbs adjusted for height, and verified that a greater ILD severity was a significant predictor of lower SMI. Recently, pectoralis muscle area (PMA) was also associated with severity and a negative annual slope of PMA related with all-cause mortality in ILD (29). Reduced quadriceps strength has been shown to be an independent predictor of exercise capacity and more studies on the therapeutic response to muscle training are needed in this population (30).

Respiratory muscle strength may also have an impact on exercise performance (31). In sarcoidosis patients with no signs of skeletal muscle involvement, twitch mouth pressure during inspiration was a stronger predictor of distance walked during 6MWT and dyspnea than respiratory function tests or oxygenation (32, 33). Given the potential effects of physical exercise, conducting randomized clinical trials including exercise-based rehabilitation seems to be a promising field for further research. However, the interrelationship between muscle dysfunction on long-term clinical outcomes (i.e., exercise capacity, dyspnea, quality of life and mortality) need to be further explored.

## PR Delivered in ILDs

Pulmonary rehabilitation (PR) for patients diagnosed with ILD are delivered in different settings and models. Traditionally, PR programs are center-based and shown positive outcomes in sarcoidosis (34) and pneumoconiosis patients (35). IPF patients have improved maximum exercise capacity and reported less shortness of breath and better quality of life after rehabilitation (36). Home-based programs are a convenient option, since they are effective, simple and an alternative for ILD patients who have restrictions accessing rehabilitation centers (37–39); moreover, this delivery model enable the interaction in order to propose changes in home environments, train family members and caregivers, as well as trying to develop skills in the patients' daily routine (37).

Most PR programs for ILD patients have a duration of 8 to 12 weeks and promote significant effects on functional capacity, quality of life and sensation of dyspnea (30, 40–46). On the other hand, short-term studies in sarcoidosis patients admitted for rehabilitation have shown benefits as reduced levels of fatigue, anxiety, depression and quality of life (34). Recently, in the face of the COVID-19 pandemic, telerehabilitation has been gaining prominence, as this strategy is safe and presents results similar to the rehabilitation programs traditionally performed in chronic respiratory disease (47), mainly in relation to the improvement of functional capacity (48). In regard to supervision, most of the studies were conducted as supervised, and higher level of improvement has been shown (6MWT) (49) in comparison with unsupervised ones, which should therefore be interpreted with caution in generalizing these findings. Besides, the effects of the intervention on exercise tolerance, health status and muscle strength are maintained after 1 year of follow-up (30).

## Exercise Prescription in ILDs

There are not many randomized and controlled studies that address physical exercise on PR for various types of ILDs. Table 1 shows the prescription of exercise-based PR in ILD patients according to eight randomized controlled trials. Most studies specifically evaluated IPF patients (62.5%) (40–44) and all studies adopted aerobic training associated with strength training as the main intervention. Only one study was conducted involving inpatients (45). In the seven studies with outpatients (30, 40–44, 46), the duration of PR ranged from 8 to 12 weeks, however most studies performed it twice a week (40–44, 46). The intensity of training aerobic training was based on previous 6MWT (30, 40, 42–44, 46), heart rate (41), oxygen consumption (45), peak work rate (30, 44) and Perez-Bogerd et al. (30) have also considered Borg scale scores to increment workload of the training. Meanwhile for strength training, some studies (30, 43, 45) prescribed the exercise intensity based on repetition maximum and, peripheral muscle training was performed with a variation of 1, 2, and 3 sets of 8, 10, 12, and 15 repetitions (30, 41, 42, 44, 45); only one study described the strength training according to time (40).

**Table 1:** Randomized controlled trials involving exercise-based pulmonary rehabilitation in patients with interstitial lung disease.

**Authors, year**	**Sample, age** **(years)**	**Setting**	**Exercise/** **functional tests**	**Intervention**	**Duration, frequency and intensity**	**Effect of intervention**	**Adverse effect/adherence**
Perez-Bogerd et al. (30)	ILD, *n* = 60 CG: 64 ± 8 ET: 64 ± 13	Outpatient	6MWT	CG: medical care ET: cycling, treadmill walking, arm cranking, stair climbing and peripheral muscle training + patient education.	60 sessions, 3x/week first 3 months and thereafter 2x/week, 90'. Cycling at 60–85% of the initial maximal work rate on the cycle ergometer and walking at 75–110% of their maximal walking speed during 6MWT + stair climbing (1–3 repetitions of 2′), peripheral muscle training → 70% of 1RM (3 sets of 8 rep).	PR improves exercise tolerance, health status and muscle force in ILD. The benefits are maintained at 1-year follow-up. The intervention did not change physical activity.	Measured/ Measured
Nishiyama et al. (40)	IPF, *n* = 28 CG: 64.5 ± 9.1 ET: 68.1 ± 8.9	Outpatient	6MWT, Cycle ergometer test	Treadmill + strength training + educational lectures.	8 weeks, 2x/week Treadmill (80% of the patient's maximal walking speed assessed at the 6MWT or 80% of the initial maximum workload at the cycle ergometer + strength training (20′).	PR improves functional exercise capacity and health-related quality of life in patients with IPF.	Not measured/ Not reported
Gaunaurd et al. (41)	IPF, *n* = 21 CG: 71 ± 6 ET: 66 ± 7	Outpatient	6MWT	Educational lectures + supervised aerobic training and strength training + home-based program (on days they did not do PR).	12 weeks, 2x/week (supervised ET) 90′+ 2 × /week (home-based program), 10 education sessions 30′ cardiopulmonary endurance training (70–80% of the maximum predicted heart rate), 20′ flexibility exercises, and 25′ strength training (initial: 2 sets of 10 repetitions; progression: 3 sets of 15 repetitions).	A 3-month PR significantly improved symptoms (SGRQ-I) and physical activity levels (IPAQ) in subjects with IPF while they actively participate in the program.	Not measured/ Not measured
Jackson et al. (42)	IPF, *N* = 21 CG: 66 ± 7 ET: 71 ± 6	Outpatient	6MWT, Cycle ergometer test	Educational lectures + cardiopulmonary aerobic training, strength training + flexibility exercise.	12 weeks, 2x/week, 120′; educational lectures (1 session biweekly) 30′ cardiopulmonary endurance training (up to 80 % maximum heart rate), flexibility exercises (3 sets x 30″) and 15–30′ strength training (up to 3 sets ×15 repetitions).	PR effectively maintained exercise oxygen uptake over 3 months and lengthened constant load exercise time in patients with moderately severe IPF.	Unclear/Unclear
Dowman et al. (43)	ILD, *n* = 142 IPF: CG: 73 ± 9 ET: 70 ± 10 Asbestosis: CG: 72 ± 9 ET: 72 ± 7 CTD-ILD: CG: 65 ± 11 ET: 63 ± 10	Outpatient	6MWT	CG: phone calls for support ET: supervised exercise program → aerobic training, cycling and walking, plus upper and lower limb strength training + home-based program.	8 weeks, 2x/week, 30′ Initial intensity for walking: 80% of peak walking speed of the 6MWT; cycling at 70% of the maximum work rate estimated from the 6MWT and, strength training at an initial load (10–12 RM). Exercise progressed weekly.	ET promoted improvement in 6MWD, symptoms and HRQoL. Magnitude of change was greater in those with asbestosis compared with IPF. Individuals with a range of severity stand to benefit, however longer-lasting effects may occur in milder disease.	No adverse event/Measured
Vainshelboim et al. (44)	IPF, *n* =3 4 CG: 66 ± 9 ET: 68.8 ± 6	Outpatient	6MWT, 30-S Chair- Stand to Test Leg Strength, CPET	CG: medical care ET: supervised exercise program → aerobic training interval + upper and lower limb strength training + flexibility exercises.	12 weeks, 2x/week, 60′ First 6 weeks: 50–60% of peak work rate in cycling and, 70–80% of individual average walking speed measured during the 6MWD and moderate intensity to strength training (1 set of 12-15 repetitions). Last 6 weeks: duration maintained, 60–70% of peak work rate in cycling and 80–90% of individual average walking speed, strength and flexibility training maintained and increased load+stair climbing (3–5′).	ET showed clinical outcomes were preserved at baseline levels with improvements in leg strength and HRQoL. The CG showed a trend of deterioration in the outcomes.	Unclear/Unclear
Greening et al. (45)	CRD, *n* =389 CG: 71.2 ± 10.0 ET: 71.1 ± 9.4	Inpatient	ISWT, ESWT	CG: standard care ET: supervised strength training and aerobic training + neuromuscular electrical stimulation. After discharge → unsupervised home- based program, + telephone consultations.	Walking was performed at a set walking speed predetermined by the ESWT at 85% oxygen consumption, ST (3 sets of 8 repetitions based on the 1RM, neuromuscular electrical stimulation (both quadriceps 30′ daily, symmetrical biphasic pulse at 50 Hz, pulse duration of 300 ms, 15″ on and five″ off.	Early rehabilitation during hospital admission for CRD did not reduce the risk of subsequent readmission or enhance recovery of physical function following the event over 12 months.	Unclear/ Measured
Holland et al. (46)	ILD (60% with IPF), *n* = 57 CG: 67 ± 13 ET: 70 ± 8	Outpatient	6MWT, CPET	CG: phone calls for support ET: supervised and unsupervised exercise program → aerobic training (cycling and walking training) + upper and lower limb strength training.	8 weeks, 2 × /week, 30′ Initial intensity for walking: 80% of peak walking speed achieved on the 6MWT.	ET improves exercise capacity and symptoms in patients with ILD, but these benefits are not sustained 6 months following intervention.	Unclear/ Measured

*ILD, interstitial lung disease; CG, control group; ET, exercise training; 6MWT, six-minute walking test; PR, pulmonary rehabilitation; IPF, idiopathic pulmonary fibrosis; SGRQ-I, St George Respiratory Questionnaire for IPF; IPAQ, International Physical Activity Questionnaire; CTD-ItD, connective tissue disease-related ILD; RM, repetition maximum; CPET, Cardiopulmonary Exercise Test; HRQoL, health-related quality of life; CRD, chronic respiratory disease; ISTW, incremental shuttle walk test ESWT, endurance shuttle walk test*.

## Safety of Exercise Training for ILDs

The safety of PR in ILD patients has been poorly studied. This topic deserves attention in future clinical trials. Development of exertional hypoxemia is common in ILD patients which can lead to adverse responses (50); as premature lactic acidosis accelerating peripheral muscle fatigue and discomfort (51), reflex tachycardia and cardiac arrhythmias (52), which may even lead to fatal adverse events (53). These aspects are particularly relevant considering that a previous study demonstrated that these patients experience various periods of hypoxemia with arrhythmogenic responses during the performance of daily activities (52). In addition, there is concern regarding the safety of delivering supplemental oxygen in patients with mild and severe hypoxemia (54). This concern is based on the accumulation of evidence about the role of oxidative stress in ILD pathogenesis (55), which can be exacerbated by physical training (56) and/or oxygen supplementation (57). On the other hand, effectiveness of oxygen supplementation to correct hypoxemia during exercise is limited. For instance, Dipla et al. (58) demonstrated that the correction of using an oxygen-enriched air (FiO_2_ = 0.40) via a Venturi mask prevented the decline in brain oxygenation, improved muscle oxygenation, and lessened dyspnea in patients with idiopathic pulmonary fibrosis (IPF) with isolated exertional desaturation. Another study demonstrated that 40% of patients with ILD, who performed exercise with the use of a pulse demand oxygen delivery device, were not able to maintain oxygen saturation >88% (59).

In this context, a previous study (36) have observed that during the period of PR and follow-up, it was observed that there were cases of deaths; however, little has been discussed about who this subgroup was, whether they were more hypoxemic or more severely impaired in their lung function. These concerns may be particularly relevant in those patients undergoing home rehabilitation or telerehabilitation (60). Additionally, a recent meta-analysis considered the main limitation was the total absence of exacerbations and adverse events reported during rehabilitative programs in the majority of studies published (35). Considering that adverse outcomes are extremely varied, it is expected that in the future, new studies will focus on more objective criteria such as: the number of patients who were unable to maintain saturation above 88% during exercise sessions (even using oxygen supplementation), electrocardiographic abnormalities (in particular, the appearance of atrial and/or ventricular arrhythmias during or after exercise) (61), amount of oxygen administered, levels of dyspnea reported during sessions, as well as their relationship with possible adverse events, such as: exacerbations, need for medication changes and new visits to the doctor, number of hospitalizations, cardiovascular events (62), fractures, skeletal muscle injures, and deaths during the study (36). These outcomes are essential to assess with more precise and objective criteria on the safety of the different models of rehabilitation programs, as well as on the impact on the follow-up of these patients. Therefore, a more complete assessment of the safety outcomes in structured programs should be the object of future studies, given its great relevance regarding the prognosis of these patients. Finally, the long-term safety in addition to potential side effects of oxygen delivery during and after an exercise training program still needs to be assessed (54).

## Adjuncts to Exercise-Based PR for ILDs

Adjunct therapies have been used during exercise-based PR of ILD patients. Currently, oxygen therapy should be prescribed for ILD patients who exhibit significant oxygen desaturation during training (<85%) (63) impacting exercise performance (64). Therefore, oxygen therapy could slow down the ventilatory limitation to exercise without altering pulmonary function or the maximal ventilatory capacity (65), which was able to promote an improvement on dyspnea (intensity and qualitative dimensions) and exercise performance in fibrotic ILD (66), and a reduction in arterial oxyhemoglobin saturation and lactic acid in ILD patients in general (67). Preliminary evidence, in fibrotic ILD patients with isolated exertional hypoxia, showed that use of portable ambulatory oxygen for 2 weeks could be an effective intervention to improvement of health-related quality of life and reduce hypoxemia and dyspnea during exertion (68). High-flow nasal cannula therapy, that manages heated and humidified air/oxygen mixtures at high flows (up to 60 L/min) (69), has been gaining popularity and is subject of recent research in patients with ILD (70–72). This technique improves ventilatory efficiency and reduces the work of breathing, generating a positive end-expiratory pressure, which may counterbalance auto-positive end-expiratory pressure, improving the oxygenation and increasing airway patency during expiration (69), being an interesting option during PR; although its benefits to exercise capacity have been proven in this population (70, 73), its effectiveness in relation to standard oxygen therapy needs further clarifications.

Non-invasive ventilation is another adjunct option during PR (74–76). Moderno et al. (75) evaluated ILD patients during three submaximal exercise tests (60% of maximum load) in three different situations: with proportional assist ventilation, with continuous positive airway pressure and without ventilatory support; the results suggest that patients on proportional assist ventilation had greater increased exercise tolerance and reduction in oxygenation and dyspnea. In addition, preliminary data (74) showed that patients with chronic hypercapnic ILD were able to benefit from nocturnal non-invasive ventilation and thus increase their physical performance and health-related quality of life. These preliminary results suggest that non-invasive ventilation may increase acutely exercise tolerance, decrease dyspnea, cardiac effort and improve the quality of life in this population. However, the chronic effects of this type of intervention on exercise responses, as well as the combination with PR, need further research.

Another adjunctive intervention for use within rehabilitation programs is neuromuscular electrical stimulation (NMES), which may be an effective treatment for muscle weakness in adults with advanced chronic respiratory disease, such as ILD (77). This modality applied prior to endurance training in patients with cystic fibrosis and severe pulmonary obstruction, is able to strengthen the peripheral muscles, improves the quality of life, reduce the ventilation required during exertion and improve the insulin resistance (78). A previous report (79) demonstrated that NMES applied on bilateral quadriceps femoris, at 40 Hz/20 min for 3 days/week during 6 weeks, associated with respiratory exercises was able to improve functional exercise capacity and prevent quadriceps weakness in ILD patients. Although adjunctive therapy is an important component of PR, more studies are necessary to define the best option related to cost, short- and long-term clinical effectiveness and prognosis.

## Conclusions and Potential Future Developments of Exercise-Based PR for ILDs

PR is an important part of comprehensive care for ILD patients in a similar way to chronic obstructive pulmonary disease patients (63). Despite evidence of the benefits of PR in ILD, this field has yet to evolve. Important issues yet to be addressed include methods to extend the duration of the benefit; and how PR can best be adapted to the complex needs of people with ILD (30). In order for these patients to benefit from the effects of PR, some barriers and facilitators need to be discussed (80).

The main barriers to the participation of patients with ILD in PR or even to the practice of physical exercises are non-adherence due to lack of understanding and the low benefits perceived by non-respondents (67). In this context, identifying the associated comorbidities, the heterogeneous clinical course of ILD and the limitations of physical exercise capacity in the physical evaluation of these patients can minimize these barriers. It is also worth mentioning that, many times, these patients are not referred to rehabilitation centers, or even have mobility and/or financial difficulties, related to the cost of transportation, especially if the individual is a home user of oxygen (80).

It is also important to consider individual aspects as barriers, such as the age of the patient, who are often patients who work, the very lack of access, the need to prescribe oxygen therapy and the difficulty of following a physical rehabilitation program (81). Another aspect that directly influences these barriers is the fear and /or lack of knowledge of family and friends concerning the physical and emotional limitations of the patient with ILD.

On the other hand, given the positive impact of PR, efforts should be made to ensure that PR is widely available across the spectrum of ILD. As facilitators, it would be important to include strategies to improve the quality of information to patients and education of medical professionals on the importance and benefits of PR, which could improve adherence rates.

It is also important to include strategies to improve the selection of these patients and more health education for patients, families, and professionals on the adequacy of referrals to avoid non-adherence to PR. Education about the importance of routine and/or self-care of physical exercises has been considered an important facilitator, however, the inclusion of this routine is influenced by the family and professional lives of individuals.

No less important, we have to propose facilitators regarding low-cost strategies and alternatives for carrying out physical training in a PR as well as maintaining the benefits achieved, considering the lack of rehabilitation centers to meet the demand of patients with chronic respiratory diseases in several regions and municipalities, mainly in the interior cities (41, 69). In addition to the limitation of displacement, due to distance, physical tiredness, the limitation of mobility imposed by the disease, hindering its availability and access.

Finally, barriers and facilitators involve physical, psychological, social, and motivational issues that require planning health costs so that the PR can offer integrated care to a larger number of patients and thus strengthen the recommendations for physical training as a central component of PR for patients with ILD.

## Author Contributions

RGM and AB-S: study conception and design. RGM, VC-S, RT, AG-A, ALGDS, SD, VAPDL, BA, and AB-S: analysis and interpretation of results and draft manuscript preparation. All authors reviewed the results and approved the final version of the manuscript.

## Funding

This work was supported by Fundação de Amparo à Pesquisa do Estado de São Paulo, N° do Processo FAPESP: 2015/26501-1 (Projects financer) and Capes finance code 001 (scholarship of VC-S and BA).

## Conflict of Interest

The authors declare that the research was conducted in the absence of any commercial or financial relationships that could be construed as a potential conflict of interest.

## Publisher's Note

All claims expressed in this article are solely those of the authors and do not necessarily represent those of their affiliated organizations, or those of the publisher, the editors and the reviewers. Any product that may be evaluated in this article, or claim that may be made by its manufacturer, is not guaranteed or endorsed by the publisher.

## Abbreviations

6MWT: 6-minute walk test
CG: control group
COPD: chronic respiratory disease
CTD-ILD: connective tissue disease-related ILD
ET: exercise training
ESWT: endurance shuttle walk test
HRQoL: health-related quality of life
ILD: interstitial lung disease
IPAQ: International Physical Activity Questionnaire
IPF: idiopathic pulmonary fibrosis
PR: pulmonary rehabilitation
RCT: randomized controlled trial
RM: repetition maximum
SGRQ-I: St George Respiratory Questionnaire for IPF.
